# A four-step implementation framework for professionalism competency in undergraduate medical education

**DOI:** 10.3389/fmed.2026.1928224

**Published:** 2026-09-17

**Authors:** Munawar Farooq, Milos R. Ljubisavljevic, Mohi Eldin Magzoub, Saeeda Al Marzooqi, Tallal Younis

**Affiliations:** 1 Department of Internal Medicine, Trauma and Emergency Research Group, College of Medicine and Health Sciences, UAE University, Al Ain, United Arab Emirates; 2 Department of Physiology, College of Medicine and Health Sciences, United Arab Emirates University, Al Ain, United Arab Emirates; 3 Department of Medical Education, College of Medicine and Health Sciences, United Arab Emirates University, Al Ain, United Arab Emirates; 4 Department of Pathology, College of Medicine and Health Sciences, United Arab Emirates University, Al Ain, United Arab Emirates; 5 Department of Internal Medicine, College of Medicine and Health Sciences, United Arab Emirates University, Al Ain, United Arab Emirates

**Keywords:** assessment, competency-based medical education, curriculum, faculty development, professional identity formation, professionalism, remediation, undergraduate medical education

## Abstract

Professionalism is a core competency in undergraduate medical education, yet many schools still rely on implicit teaching and inconsistent responses to professionalism lapses. Reports showing how institutions operationalize professionalism across standards, teaching, assessment, and learner support remain limited, especially in settings where cultural norms shape communication, accountability, family involvement, and conduct. We describe an evidence-guided, theory-informed implementation framework developed during a two-year institutional improvement cycle at the College of Medicine and Health Sciences, United Arab Emirates University. The development approach combined literature review, benchmarking, a structured nominal group process with core faculty, committee deliberation, and stakeholder review. The resulting framework comprised four steps: clear understanding, curricular integration, standardized assessment, and remediation with well-being support. It defined professionalism for physicians and translated the same construct for medical students across academic, clinical, and community settings; embedded teaching through an expectation-experience-evaluation cycle; introduced a common assessment package; and linked coaching, committee review, mentoring, and counseling within a developmental response pathway. The design drew on situated and experiential learning, reflective practice, professional identity formation, and programmatic assessment. Early implementation indicators showed broader curricular integration: professionalism moved from two early courses to a spiral curriculum, most preclinical modules include expectation-setting sessions, and longitudinal courses use reflection assignments. Professionalism is now graded within a must-pass competency framework in the first two years; problem-based learning engagement grades align with the rubric; and all clerkships use a standardized professionalism rubric that contributes 10% of clerkship marks. During the first year of implementation, approximately 60% of reported incidents were investigated by the committee; in the current phase, approximately 60% are managed at clerkship level, with fewer cases referred for committee review. The main contribution is a transferable four-step approach that schools can adapt while preserving the implementation sequence and localizing standards, learning activities, assessment anchors, and support pathways.

## 1 Introduction: background and rationale for the educational activity innovation

Physician professionalism influences patient experience, quality of care, teamwork, and physician well-being. Professionalism concerns during training are associated with later disciplinary action in practice (1, 2). Yet it remains difficult to implement professionalism coherently in undergraduate medical education. Recent reviews show wide variation in how schools define, teach, assess, and sustain professionalism, with continuing problems in faculty calibration, longitudinal follow-up, and the link between assessment and remediation (3, 4).

Medical students are expected to uphold high professional standards, including punctuality, engagement, and integrity, as they develop their professional identity and acquire the desirable attributes of medical professionalism within local and global communities of practice (5). However, unprofessional behaviors, ranging from minor lapses to serious breaches, frequently generate dissatisfaction among students, faculty, and leadership (6). This is compounded by disparities in institutional capacity and culture in addressing professionalism relative to other competencies, such as medical knowledge and clinical skills (7).

The difficulty of implementing professionalism is both organizational and educational. Professionalism is enacted across learning environments, shaped by role modeling, and interpreted through local norms around hierarchy, communication, responsibility, and relationships with patients and families. Arab-region scholarship emphasizes accountability, duty, respect, strong family bonds, and social trust, suggesting that schools should not rely on imported descriptors without local interpretation (8–10). Even so, professionalism is still often managed as a series of isolated lapses rather than as a competency requiring explicit standards, repeated practice, shared assessment language, and longitudinal support.

At the College of Medicine and Health Sciences, United Arab Emirates University (CMHS-UAEU), several converging signals prompted action. Coordinators reported inconsistent expectations and documentation for student professionalism across rotations; students appealed decisions citing unclear standards and variable application; frontline faculty had no common mechanism to distinguish concerns better handled through immediate coaching from those needing formal documentation, committee review, or more structured support; and existing policy language signaled that professionalism mattered but did not provide a practical implementation system.

The college therefore approached professionalism as an implementation challenge. Our aim was to develop a reproducible framework that aligns shared standards, curriculum, assessment, and developmental response. Although developed for professionalism, the same framework sequence can be adapted to other complex competencies in undergraduate medical education.

## 2 Pedagogical framework, learning theories, principles, and competency standards underlying the educational activity

The framework comprises four linked steps: clear understanding, curricular integration, standardized assessment, and remediation with well-being support (Figure 1). Its design was informed by educational perspectives commonly used in medical education. Situated and social learning emphasize that professionalism is learned through participation in authentic academic and clinical work, observation of role models, and gradual entry into a community of practice rather than through decontextualized teaching alone (11, 12). Experiential learning and reflective practice support repeated cycles of action, feedback, and reflection so that learners can make sense of ethically and emotionally complex encounters (13, 14). Professional identity formation provides the developmental lens linking role modeling, mentoring, reflection, and longitudinal follow-up (15).

**Figure 1:**
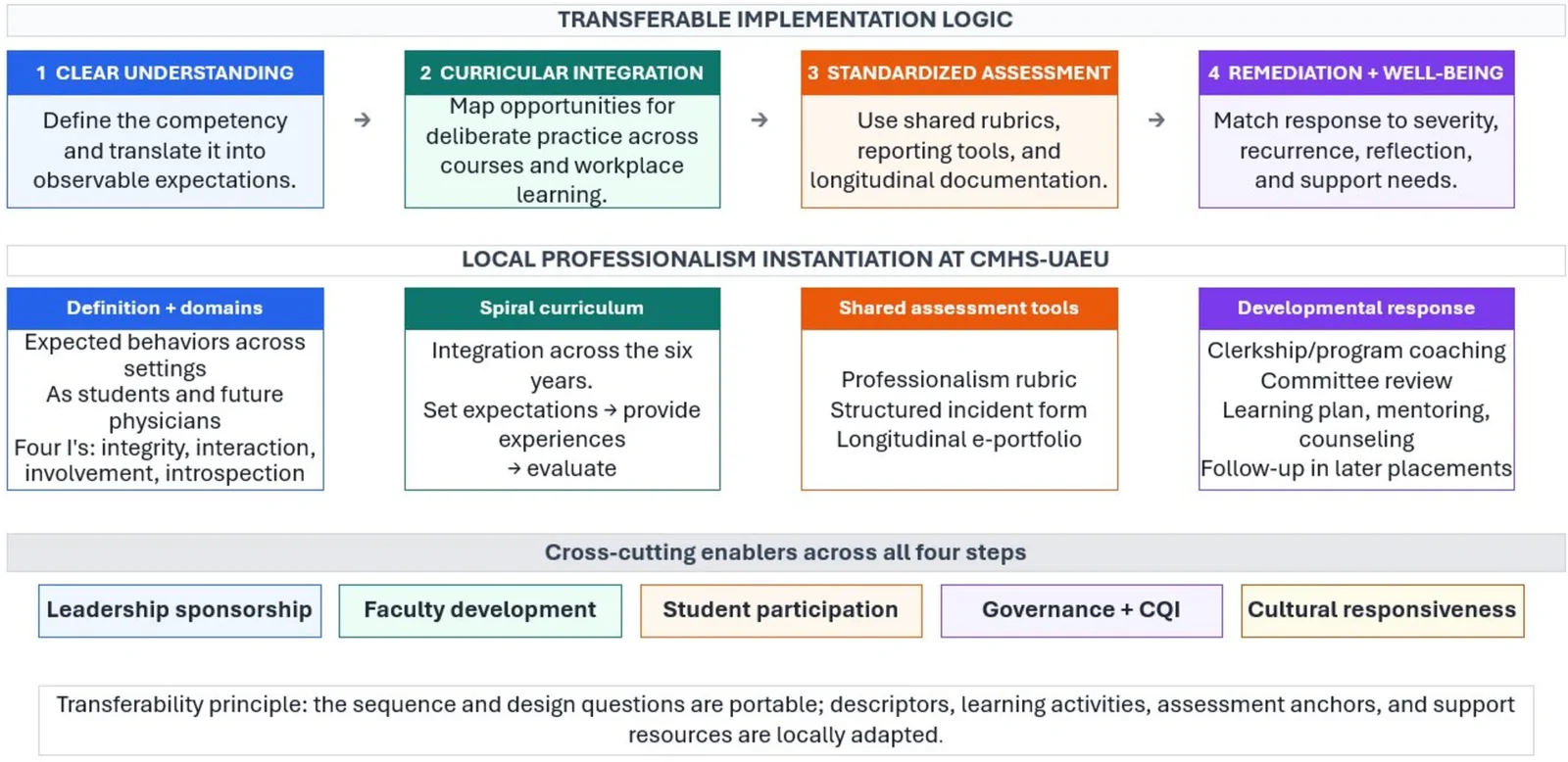
Four-step implementation framework for professionalism competency in undergraduate medical education.

These perspectives shaped the logic of the framework. Clear understanding provides conceptual and normative framing; curricular integration creates authentic opportunities to enact the required competency; standardized assessment aggregates observations across settings; and remediation with well-being support turns assessment into coached development. The framework, therefore, moves from defining the construct to supporting its enactment, interpretation, and improvement.

For the first step, we adopted a working definition of medical professionalism as the values, behaviors, and relationships that society expects from physicians in caring for patients and families, working with colleagues and staff, and regulating themselves in ways that sustain trust. For educational use, the same construct was adapted for medical students as the expected behaviors, responsibilities, and relationships of learners in academic, clinical, and community settings, appropriate to their level of responsibility and contact with patients and families. To translate this definition into observable expectations, the Student Professionalism Committee adopted the “4 I” model from the literature, which organizes concerns and strengths into four operational domains: integrity, interaction, involvement, and introspection (16). Together, the definition and domains gave faculty and students a shared vocabulary for behaviors that are often difficult to express explicitly.

The second step treated professionalism as a longitudinal classroom and workplace competency. Teaching was embedded across orientation, preclinical courses, simulation, early clinical exposure, longitudinal courses, and clerkships using an expectation-experience-evaluation cycle. Expectations were stated explicitly; authentic activities created opportunities to practice the competency; and feedback, reflection, and assessment made learning visible. This structure reflects experiential and situated learning by placing professionalism within ordinary educational work and by using role modeling, participation, and guided reflection to address both the formal and hidden curricula (5, 11, 13, 14).

The third step focused on shared assessment. The college introduced a professionalism assessment package comprising a common rubric aligned with the four domains (integrity, interaction, involvement, and introspection) and a structured event-based report form. The rubric provides domain ratings and an overall performance level from 5 (good) to 1 (extreme), with an NA option. Routine observations, rubric ratings, narrative feedback, incident reports, and e-portfolio records were treated as complementary data points. This design aligns with programmatic assessment: individual observations are primarily used for feedback and documentation, whereas interpretation depends on patterns across time, settings, and raters (16, 17).

The fourth step emphasized developmental response. Lower-level or isolated concerns were expected to prompt direct coaching and clarification of expectations. Repeated, serious, or poorly responsive concerns could be escalated for committee-based review, documented learning plans, mentoring, counseling, or further monitoring. A conscious and deliberate effort was maintained to protect student well-being during the remediation process through engagement of mentoring and counseling programs. This approach treated remediation as part of competency development rather than as a purely disciplinary process and linked response intensity to severity, recurrence, reflectiveness, and adaptability (18).

Together, the four steps constitute the transferable implementation sequence; competency-specific standards, examples, learning activities, assessment anchors, and support pathways require local adaptation (Table 1).

**Table 1:** Transferable elements of the four-step framework and their local application to professionalism.

Framework step	Transferable institutional task	Local professionalism example
1. Clear understanding	Define the competency in observable terms and translate it into shared expectations for faculty and learners.	Shared definition for physicians and students; Student Professionalism Guidelines; four operational domains: integrity, interaction, involvement, and introspection.
2. Curricular integration	Create repeated opportunities to practice the competency across phases of training using explicit expectation-experience-evaluation cycles.	Orientation, preclinical expectation-setting sessions, longitudinal reflection assignments, simulation, early clinical exposure, and clerkships.
3. Standardized assessment	Use common tools, documentation rules, and longitudinal records so the competency can be judged consistently across settings.	Shared rubric, structured report form, routine professionalism score reporting in all clerkships, and e-portfolio follow-up.
4. Remediation and well-being	Match response intensity to pattern and severity while linking feedback to support resources and follow-up.	Local coaching for lower-level concerns; committee review for repeated or serious concerns; mentoring, counseling, and learning plans.

To adapt the framework to another competency, preserve the transferable task in column 2 and replace the professionalism-specific content in column 3 with locally defined standards, learning experiences, assessment anchors, and support pathways.

## 3 Learning environment (setting, students, faculty); learning objectives; pedagogical format

The framework was developed and implemented at the College of Medicine and Health Sciences (CMHS), United Arab Emirates University (UAEU), Al Ain, United Arab Emirates. CMHS is a public, undergraduate-entry medical school and one of the country's oldest medical colleges, with a national role in preparing Emirati physicians. Admission is limited to UAE nationals, and the student body is predominantly female, with an approximate male-to-female ratio of 1:3. The college graduates slightly more than 100 physicians each year, consistent with a recent study describing it as the country's largest medical school (10). The program spans six years followed by a mandatory internship year. The first two years are premedical and include basic sciences, general education, and professionalism-related courses; years 3 and 4 are preclinical, with system-based pathology and clinical skills learning supported by simulation; and years 5 and 6 are clinical, during which students complete clerkships in affiliated healthcare settings (10, 14). Before this framework, professionalism was taught mainly through two courses in the premedical phase and was not consistently articulated as an explicit longitudinal competency across subsequent courses and clerkships. Nor was professionalism routinely assessed or graded as a separate component in end-of-course or clerkship assessments. Professionalism concerns were managed largely through unprofessional incident reports; however, the absence of shared guidelines and assessment anchors made expectations difficult to communicate and led to frustration for both students and faculty. This institutional context therefore provided a strong educational rationale for a framework that could make professionalism visible, teachable, assessable, and supportive across the whole program.

Institutional sponsorship came from the Undergraduate Medical Curriculum Committee, which charged a faculty group from the preclinical program, clerkship program, and medical education unit with creating a workable implementation system. A Student Professionalism Committee supported governance, review, and continuity and included leadership and faculty from the preclinical and clinical programs, medical education, student counseling and mentoring, and a student representative. Course coordinators, program directors, and frontline faculty implemented expectation-setting, routine observation, feedback, assessment, and first-line management; the committee oversaw escalation and longitudinal review, while existing mentoring and counseling services supported remediation. Implementation was embedded in existing courses, clerkships, committee structures, and the e-portfolio.

The objectives of the innovation were fivefold: first, to create a clear and culturally responsive institutional definition of professionalism for both physicians and students; second, to embed professionalism teaching longitudinally across undergraduate medical education; third, to standardize how professionalism is assessed, documented, and escalated; fourth, to create a structured response system that links remediation to mentoring and well-being support; and fifth, to build a framework whose implementation logic could later be adapted to other competencies.

The pedagogical format combined evidence-guided design with participatory refinement. The faculty group first conducted a narrative review and benchmarked regional and international approaches to professionalism, assessment, remediation, and student guidance. A structured nominal group process was then undertaken with five senior faculty members representing the preclinical program, clinical program, and medical education (19, 20). The group met three times and continued its work between meetings through online communication. Members generated, clarified, prioritized, and refined the professionalism domains, behavioral descriptors, assessment rubric, event-based reporting form, and response pathway. Before the guidelines were finalized, the draft was circulated by email to wider faculty, students, and institutional leadership for feedback; comments were reviewed and incorporated where appropriate. The resulting Student Professionalism Guidelines are provided as Supplementary File 1.

Faculty development was essential to implementation. Two shared webinars helped establish a common language for faculty and students, featuring key presentations from both students and faculty. Annual clerkship retreats involving more than 20 clinical faculty focused on how to teach, observe, and respond to professionalism in routine practice. Faculty were encouraged to promote professionalism as teachers, assessors, and role models and to use the expectation-experience-evaluation cycle in courses and clerkships. A workshop for clerkship coordinators focused primarily on how to approach a learner in difficulty.

Student participation occurred through webinars, guided discussions, and reflective assignments linked to academic and clinical experiences. Reflection was used to help students identify and interpret conflicts between the formal and hidden curricula, including messages conveyed through daily routines, workplace interactions, and institutional responses (5, 13–15, 21). Teaching in the Emergency Medicine clerkship provided one example of this approach, with students reflecting on observed encounters, role modeling, and tensions between stated ideals and real-world practice (14).

Operationally, professionalism expectations were introduced at orientation and reinforced at the start of relevant modules and clerkships. Faculty observed students during routine activities and provided prompt, individualized feedback, while coordinators incorporated professionalism into scheduled performance reviews. Minor concerns were addressed through clarification and coaching; persistent, serious, or unresolved concerns were documented using the incident report, triaged by course or program leaders, and referred to the Student Professionalism Committee when indicated. The committee coordinated appropriate remediation, mentoring, counseling, and longitudinal follow-up. Implementation was monitored through quarterly committee meetings, with additional *ad hoc* meetings as needed, and annual clerkship retreats; these forums reviewed curricular coverage, rubric use, incident outcomes, and e-portfolio documentation.

## 4 Results to date and assessment

The evaluation strategy tracked curricular reach, use of common assessment and documentation tools, incorporation of professionalism into progression decisions, disposition of reported concerns between course/clerkship and committee levels, and descriptive relationships between professionalism scores and at-risk academic performance. The initial cycle produced a coherent set of structures and tools: a shared definition, Student Professionalism Guidelines, regular committee proceedings, a common rubric, a structured report form, and longitudinal e-portfolio documentation. Figure 1 illustrates the four-step implementation framework developed for professionalism competency in undergraduate medical education. The upper row shows the transferable implementation logic; the lower row shows the local professionalism-specific content. Cross-cutting enablers operate across all four steps.

Curricular integration broadened substantially. Initially, professionalism was addressed mainly through two premedical courses during the first two years; it is now organized as a longitudinal, spiral curriculum across all years. Students in the premedical program submit written self-reflections twice each year. In years 3 and 4, introductory sessions in preclinical modules explicitly follow the expectation-experience-evaluation sequence. Problem-based learning engagement grading in preclinical modules was aligned with the standardized professionalism rubric. Longitudinal courses incorporated structured professional reflection assignments.

Assessment became more visible and consequential. Professionalism is now assigned 10% of total marks in longitudinal courses within a must-pass competency framework. In the clinical phase, all clerkships use the four-domain rubric and attribute 10% of clerkship marks to professionalism. The final 10-point component is not a mechanical conversion of the 1–5 rubric or a count of incidents; coordinators apply rubric-informed academic judgment to the pattern, severity, recurrence, affected domains, insight, responsiveness to feedback, reflection, and adaptability. Together, these practices shifted professionalism from an optional or individually variable concern to a routinely assessed and documented component of student progression.

The response pathway also matured. Figure 2 illustrates the pathway for professionalism assessment and remediation as provided in the Student Professionalism Guidelines. Before implementation, incidents were often underreported or sent directly to leadership for disciplinary action. The two-tier system allowed most concerns to be addressed within clerkship programs, with outcomes reported to the Student Professionalism Committee and escalation reserved for recurrent, serious, or poorly responsive cases. During the first year of implementation, approximately 60% of reported incidents were investigated by the committee. In the current phase, approximately 60% are managed at clerkship level, with fewer cases referred for committee review. Students undergoing remediation are monitored longitudinally through committee review of mentoring reports and performance in subsequent clerkships.

**Figure 2:**
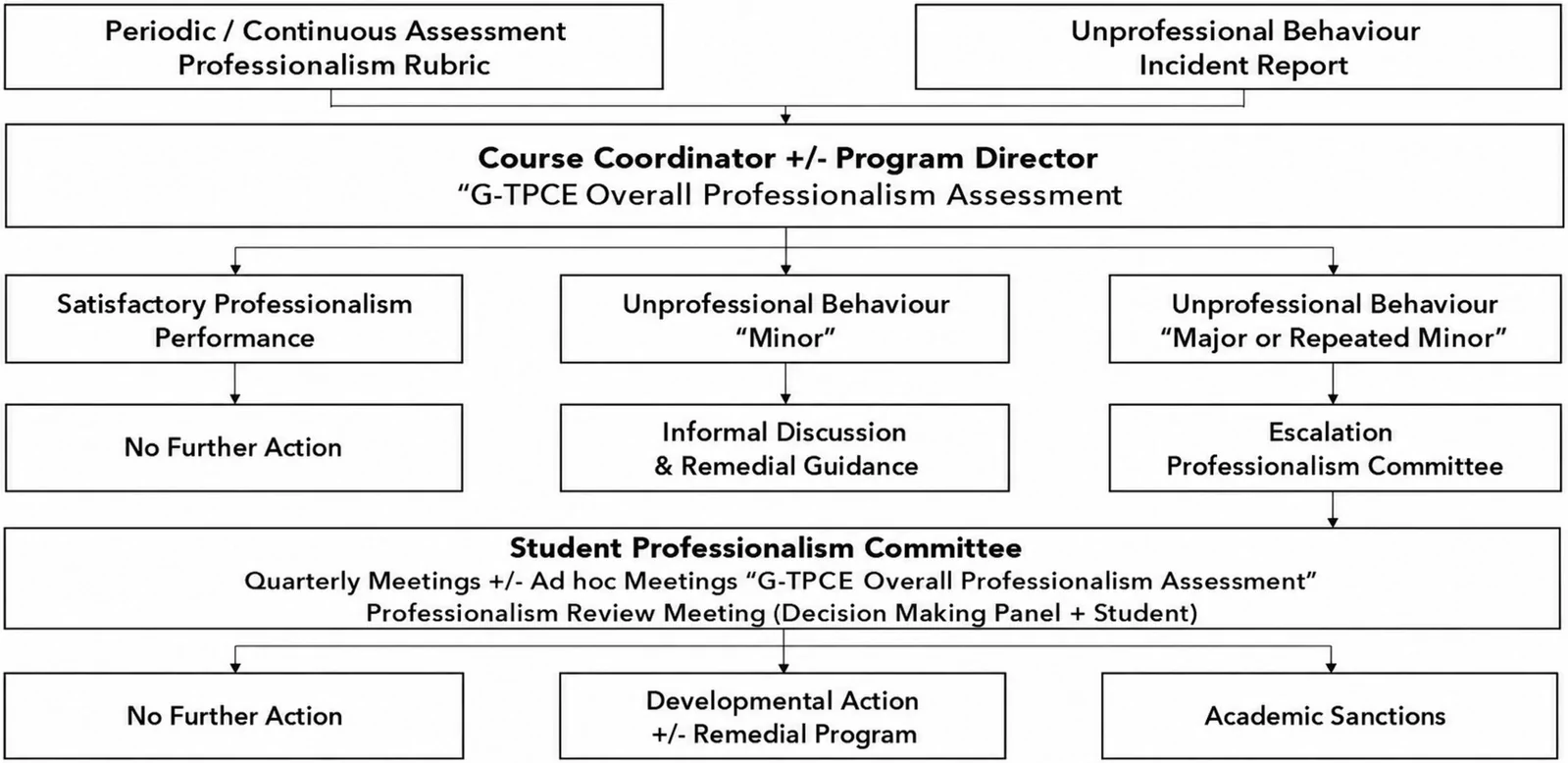
Flowchart of professionalism assessment and unprofessional behavior review process.

Figure 3 provides a descriptive assessment signal from the clinical clerkships, representing 106 students across six clerkships in each of the junior and senior years. At-risk course performance was defined as failure (<70% overall clerkship mark) or borderline performance ( 70%-<75% overall clerkship mark) in any clerkship. The professionalism component was scored out of 10 using the rubric-informed judgment described above. Overall, there were 10 reported failures and 28 borderline course performances in the junior clerkships, as well as 10 reported failures and 15 borderline course performances in the senior clerkships. Mean professionalism scores were lower among at-risk course performances than among the overall clerkship cohorts (junior clerkships: 8.50 vs. 9.63; senior clerkships: 8.66 vs. 9.55). These descriptive data suggest that students at academic risk may also require closer professionalism monitoring and support, and that professionalism should be interpreted longitudinally alongside other competency data.

**Figure 3:**
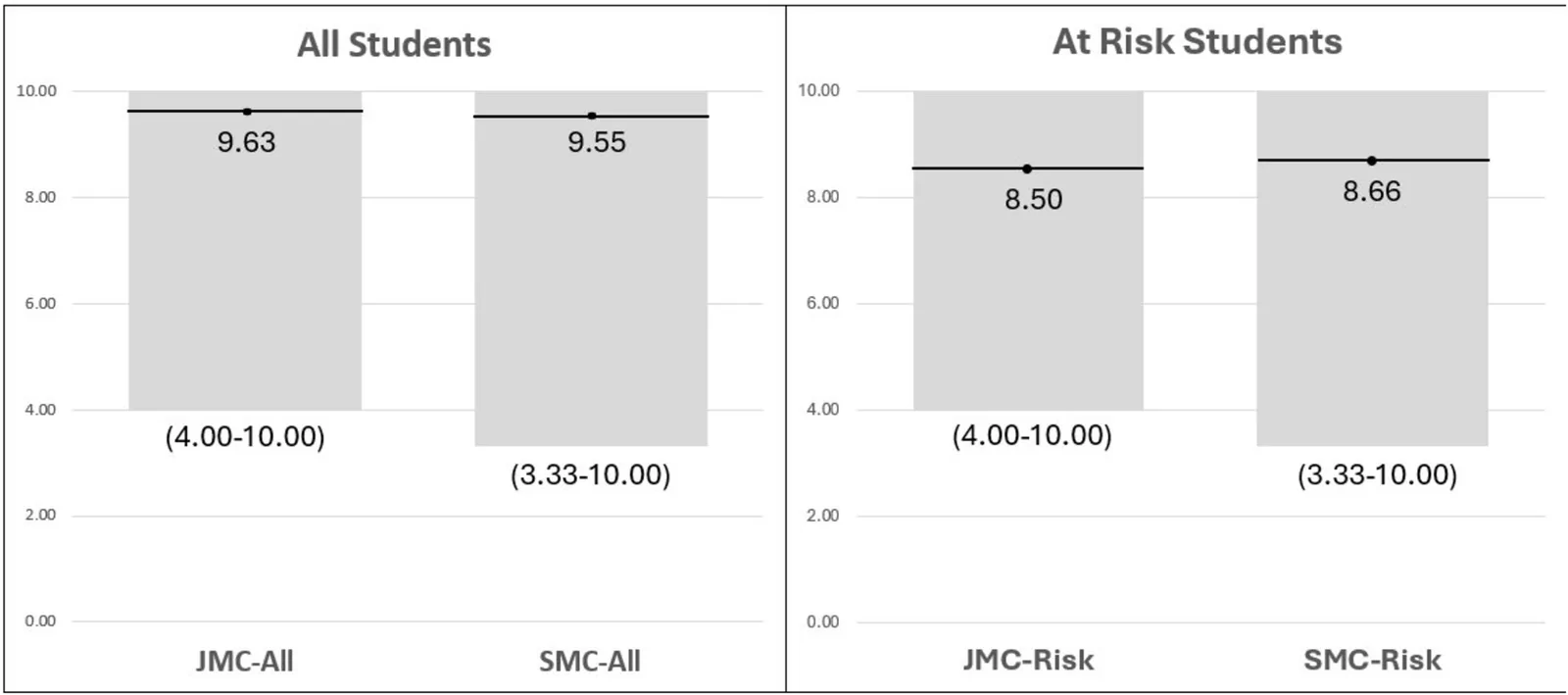
Professionalism score distributions for all students and for at-risk course performances in junior and senior clerkships. Professionalism scores were awarded out of 10 using the standardized rubric. Points show mean scores, and boxes show minimum-to-maximum ranges. At-risk course performance was defined as failure (<70% overall clerkship mark) or borderline performance (70%-<75% overall clerkship mark) in the relevant clerkship.

Together, these outputs produced three working rules for the next phase: use the expectation-experience-evaluation cycle in every course or clerkship; treat every faculty member as teacher, assessor, and role model of professionalism and professional identity formation; and review all four steps through continuous quality improvement rather than reacting only to isolated incidents. Table 1 shows the transferable elements of the four-step framework and how the same logic can be transferred to other competencies while preserving local adaptation.

## 5 Discussion: practical implications, objectives, and lessons learned

The individual elements of the initiative are established in the literature: explicit standards, longitudinal learning, structured assessment, event reporting, remediation, and professional identity support have each been described previously (3, 4, 16–18). The contribution of this work lies in their institution-wide integration into a four-step implementation architecture that uses one shared language across academic and clinical settings, links routine assessment to a two-tier developmental response with well-being support, and separates a stable implementation sequence from locally adaptable descriptors, learning activities, assessment anchors, and resources. Contextual adaptation of the professionalism standards and the operational linkage among curriculum, governance, documentation, assessment, and learner support distinguish the framework from isolated tools or courses (8–10, 22, 23).

The early indicators suggest that the framework progressed beyond tool creation into routine curricular and assessment practice: professionalism became longitudinal, graded, documented, and increasingly managed at the course/clerkship level while committee oversight remained available for escalation and follow-up.

Several lessons emerged. First, professionalism became more teachable when it was translated into shared domains and observable expectations. Second, authentic participation mattered: students learned through expected behaviors during routine classroom and clinical work, role modeling, and structured reflection on the hidden curriculum, consistent with situated and experiential learning. Third, longitudinal mentoring, feedback, and reflection supported professional identity formation rather than treating professionalism as a checklist of isolated behaviors (11, 13, 15, 18).

Assessment became more useful when it was embedded in a developmental system. The common rubric, report form, and e-portfolio created shared language and longitudinal documentation, while the response pathway connected observations to coaching, committee review, and support. Figure 3 suggests that students at academic risk may also face professionalism challenges. A comparable association between objective measures of professionalism and academic achievement has been reported among first- and second-year osteopathic medical students (24).

Transferability depends on preserving the four-step sequence while adapting the competency-specific content and institutional resources to local context (Table 1).

## 6 Constraints

This work has several constraints. It describes a single-institution implementation experience and does not present a formal outcome study of behavior change, patient-care effects, or long-term professional identity trajectories; no pre/post comparison or control group was used, and the process indicators do not establish causality. The professionalism rubric was developed with the broader Student Professionalism Guidelines through the structured nominal group process and stakeholder review, but its psychometric properties were not evaluated in this phase. The clerkship score analysis is descriptive and may be influenced by contextual and rater variation. Implementation also relied on existing governance, e-portfolio, mentoring, and counseling resources that may differ across institutions.

The next evaluation phase should examine multiyear trends in incident reporting, the proportion and nature of cases resolved at course/clerkship versus committee level, recurrence after remediation, and the association between professionalism and academic performance marks. Where feasible, it should also assess rater consistency, learner and faculty experience, and longer-term educational outcomes. Within these limits, the framework offers a practical implementation sequence that other programs can adapt.

## Data Availability

The original contributions presented in the study are included in the article/Supplementary Material, further inquiries can be directed to the corresponding author.
