# Supplementary material for: A four-step implementation framework for professionalism competency in undergraduate medical education
Source: Front Med (Lausanne). 2026 Sep 17;13:1928224. doi: 10.3389/fmed.2026.1928224 (PMC13626880; doi:10.3389/fmed.2026.1928224)
Supplement: Supplementary file 1 [file Datasheet1.pdf]

CMHS MD Program Guidelines		
**Subject**<br>MD Student Professionalism	**Guideline Number**	MD – 4
	**Effective Date**	1 – Aug – 2024
**Responsible Office:**<br>Assistant Dean for Medical Education	**Most Recent Review Date**	26 – June – 2024
	**Due Date for Next Review**	January, 2027

MD Student Professionalism Guidelines

College of Medicine and Health Sciences, CMHS, UAEU

Endorsed by CMHS College Council on June 26, 2024

Table of Contents

1. Introduction 2
2. Definitions and Scope 2
3. Core Principles of Professionalism 2
4. Standards of Professional Behavior 3
5. Roles and Responsibilities 5
6. Assessment and Evaluation of Professionalism 6
7. Professionalism Review Process 8
8. Processes for Addressing Unprofessional Behavior 14
9. Guidelines Review and Amendments 15

Appendix 1: Professionalism Rubric 17

Appendix 2: Illustrative Cases and Examples of unprofessional behavior 19

Examples of unprofessional behavior 22

Appendix 3: Unprofessional Behaviour Report Form 24

Appendix 4: Cultural Sensitivity 25

Appendix 5: Bibliography 26

1. Introduction

Welcome to the Student Professionalism Guidelines of the College of Medicine and Health Sciences at UAE University. These guidelines foster a culture of excellence, respect, and ethical behavior among our medical students. They define the expectations for professional conduct in academic, clinical, and digital settings and are integral to the development of students as future healthcare providers.

As future physicians, students are expected to uphold the highest standards of integrity and respect in all interactions with peers, faculty, patients, and the community. These guidelines serve as a roadmap to help navigate the challenges and expectations of a professional medical career within the UAE's culturally rich and diverse landscape.

2. Definitions and Scope

- **Professionalism**: Within the College of Medicine and Health Sciences, professionalism consistently demonstrates core ethical values through personal behavior and interactions in all settings. These values include, but are not limited to, altruism, accountability, excellence, duty, honor, respect, and sensitivity.
- **Scope of Application**: These guidelines apply to all aspects of a student's academic and clinical training, digital interactions, and any professional settings associated with their training. This broad scope ensures that professionalism is maintained during all forms of interaction, including:
    - **Academic Settings:** Students must demonstrate integrity in examinations and assignments, actively participate in learning activities, and engage respectfully with faculty and peers. This includes adhering to the academic honor code and collaborating with classmates in a supportive manner.
    - **Clinical Settings:** Professionalism in clinical environments involves maintaining patient confidentiality, obtaining proper consent, interacting respectfully with patients and staff, and demonstrating appropriate clinical conduct. Students should be particularly mindful of cultural sensitivities when interacting with patients from diverse backgrounds and adhering to dress codes that respect the cultural norms of the UAE.
    - **Digital Presence:** Professionalism extends to students' digital presence, where they are expected to maintain the same standards of respect and integrity as in face-to-face interactions. This includes appropriate use of social media, email communications, and other online platforms, ensuring that all digital interactions are conducted with professionalism and respect for privacy and confidentiality.

3. Core Principles of Professionalism

CMHS's core principles of professionalism are designed to foster trust, respect, and integrity in all professional and personal interactions. By adhering to these principles, students will contribute positively to the ethical and professional medical community, both within UAE University and in the wider world:

- **Altruism and Patient Care**: Students are expected to place the well-being of patients above their own interests. This includes providing compassionate and respectful care to all patients, regardless of their background, and preserving their dignity and rights. Demonstrating empathy and understanding of patients' cultural and personal values is crucial.
- **Integrity and Accountability**: Students' actions should be guided by honesty, transparency, and a commitment to ethical practices. This includes admitting mistakes, taking responsibility for actions, and striving to correct them. Accountability also involves being reliable in all academic and clinical duties.
- **Respect for Others**: This principle requires students to respect everyone they interact with, including patients, peers, faculty, and staff. This involves valuing diversity, embracing cultural differences, and demonstrating empathy in all interactions. Students should be particularly aware of the cultural norms and expectations in the UAE, ensuring respect in communication styles and interpersonal interactions.
- **Commitment to Excellence**: Students should aim for the highest quality in all aspects of their academic and clinical work. This involves a commitment to continuous learning and improvement, actively seeking feedback, and responding constructively to assessments. Excellence also means being prepared and punctual for all academic and clinical engagements.
- **Justice in Healthcare**: Students are encouraged to advocate for and practice fair treatment and equitable distribution of healthcare resources. This includes recognizing and addressing healthcare disparities and ensuring care is delivered without bias or discrimination. Understanding the socio-economic and cultural contexts that affect healthcare access in the UAE is part of this commitment.

4. Standards of Professional Behavior

To effectively translate the core principles into everyday practice, the following standards of professional behavior are established. These standards provide specific expectations for student conduct in various settings:

Academic Integrity

- Uphold the highest standards of honesty and integrity in all academic work. This includes avoiding plagiarism, cheating, or any form of dishonest conduct. Engage actively in learning activities, contribute meaningfully to discussions, and respect peers' learning opportunities by maintaining a collaborative and supportive educational environment.

Clinical Conduct

- Demonstrate respect and compassion for patients by maintaining confidentiality and obtaining informed consent for all medical procedures. Adhere to clinical dress codes and professional appearance standards, respecting cultural sensitivities that are particularly relevant in the UAE. This includes dressing modestly and appropriately in accordance with both professional standards and local cultural expectations.

- Display punctuality, preparedness, and professionalism in all clinical rotations and interactions with clinical staff. Be especially mindful of the hierarchical and respectful interactions expected in clinical settings in the UAE, showing deference where culturally appropriate.

Professional Conduct in Digital and Social Media

- Maintain professionalism in all online and digital interactions by respecting confidentiality and avoiding sharing inappropriate content. Use professional language in digital communications, including emails and social media posts, reflecting the decorum expected of medical professionals.
- Be cautious in the digital dissemination of information, particularly concerning patient or sensitive university materials. Adhere to guidelines that prevent the sharing of confidential or proprietary information.

Interaction with Peers and Faculty

- Foster an environment of mutual respect and courtesy in all interactions with faculty and fellow students. This includes engaging in constructive conflict resolution and seeking mediation or guidance to resolve interpersonal conflicts professionally.
- Support and contribute to a learning environment that respects diverse perspectives and encourages open dialogue. Practice effective communication skills that reflect respect and understanding for the UAE's diverse cultural backgrounds.

5. Roles and Responsibilities

To ensure the effective implementation of the professionalism guidelines, clear roles and responsibilities are defined for students, faculty, and peers:

Role of Students

- **Uphold Professional Standards:** Students are primarily responsible for upholding the standards of professionalism as outlined in these guidelines. This includes demonstrating ethical behavior, integrity, and respect in all academic, clinical, and digital interactions.

- **Active Engagement:** Students should actively engage in learning opportunities that enhance their understanding and application of professional behaviors. This involves participating in workshops, seminars, and peer reviews and utilizing the support and resources provided by the college.

- **Self-Improvement:** Students must reflect on feedback and assessments related to their professionalism, actively seeking to improve and rectify any deficiencies. They must also participate in self-assessment exercises and work with mentors or advisors to develop their professional identities.

Role of Faculty and Administrators

- **Modeling Professional Behavior:** Faculty and administrators are responsible for modeling exemplary professional behavior and providing a benchmark for students. They should embody

the core principles of professionalism in their interactions with students, colleagues, and the community.

- **Teaching and Promotion:** Actively promote and teach the principles of professionalism through the curriculum and introspect clinical settings. This includes integrating professionalism into lectures, seminars, and hands-on training and providing clear examples of professional behavior.
- **Assessment and Feedback:** Assess student professionalism fairly and consistently, providing constructive feedback to help students improve. Faculty should use the established rubrics and benchmarks to evaluate professionalism in academic and clinical settings.
- **Support and Guidance:** Offer support and guidance to students, especially those struggling with professional behavior. This includes developing remediation plans and mentoring students to help them meet professional standards.
- **Reporting Incidents:** While we respect the professional judgment of our faculty and staff, we also expect them to report observed or suspected incidents of unprofessional behavior. The guidelines provide a clear framework to help determine when and how to report these incidents, ensuring consistency and supporting our commitment to high standards of professionalism within the college.

Role of Peers

- **Supportive Environment:** Peers play a crucial role in supporting one another's professional development. They should foster a supportive and respectful learning environment where professional behaviors are practiced and encouraged.
- **Peer Feedback:** Engage in peer assessments, providing constructive feedback on professional behaviors. This process helps build a collaborative learning environment and encourages continuous student improvement.
- **Role Modeling:** Senior students and those who demonstrate strong professional behavior should act as role models and mentors to their peers, sharing their experiences and guiding others in developing their professionalism.

6. Assessment and Evaluation of Professionalism

Professionalism is a critical component of medical education at CMHS, and its assessment and evaluation are essential to fostering a culture of ethical behavior and respect. The following guidelines outline the methods and practices used to assess and evaluate professionalism among students:

Continuous Assessment

Continuous assessment provides ongoing insights into students' professional development, ensuring timely feedback and support to enhance their professional conduct.

- **Direct Observations**: Faculty and staff will observe students' professionalism in academic and clinical settings. This assessment will focus on:
  - Students' interactions with peers, faculty, patients, and staff.
    - Adherence to ethical and professional standards.
    - Communication skills, especially in sensitive or challenging situations.
    - Cultural sensitivity, focusing on respecting and adapting to the UAE's diverse cultural norms and patient backgrounds.
- **Feedback Mechanism**: Students will receive immediate and regular feedback on their professionalism, which will be:
    - **Constructive and Specific:** Aimed at guiding students to improve their professional conduct by providing specific examples and actionable advice.
    - **Scheduled Regularly:** Delivered during feedback sessions to discuss student performance and progress.
    - **Documented:** Recorded in students' personal development files to track progress.

Peer Reviews

Peer reviews are essential to developing a supportive and reflective learning environment, allowing students to learn from one another.

- **Structured Peer Assessments:** Students will engage in structured peer reviews, which will:
  - Allow them to provide and receive constructive feedback on professional behaviors using standardized forms and criteria.
  - Encourage critical thinking about their own and others' behaviors.
  - Help identify common challenges and foster a collaborative approach to finding solutions.

Self-Assessments

Self-assessment encourages students to reflect on their professionalism and identify areas for improvement.

- **Reflective Practice:** Regular self-assessment exercises will be integrated into the curriculum to:
  - Guide students in reflecting on their professional development using specific questions and criteria provided by the faculty.
  - Require students to maintain reflective journals, documenting their experiences, insights, and progress.
  - Encourage students to develop personal development plans in consultation with mentors to improve their professionalism.

Integration into Curriculum and Grading System

Professionalism is a core component of the curriculum and significantly affects students' grades, reflecting its importance in their development.

- **Grading Professionalism:** The integration of professionalism into the grading system will:

  - Be clearly communicated through specific benchmarks and rubrics provided at the beginning of each course or clinical rotation.
  - Detail the weight of professionalism in the overall grade to underscore its importance.
- **Documentation:** Comprehensive and transparent record-keeping will support the assessment process.
  - **Professionalism Portfolio:** Students will maintain a portfolio where all assessments, feedback, and incidents of unprofessional behavior are documented.
  - **Accessible and Confidential:** Students will have access to their records to review their progress while maintaining confidentiality.
  - **Review Process:** The procedures for reviewing and contesting records will be clear and fair to maintain transparency and fairness in the assessment process.

Best Practices in Assessing Professionalism

To ensure the assessment of professionalism is aligned with best practices:

- **Use Multiple Methods:** To capture the full spectrum of professional behaviors, a variety of assessment methods, including direct observation, peer review, and self-assessment, will be used.
- **Ensure Consistency:** Assessments will be consistent across different settings and times to provide a reliable measure of students' professionalism.
- **Incorporate Cultural Sensitivity:** Assessments will include elements of cultural sensitivity, reflecting the unique context of the UAE and preparing students for diverse environments in their practice.
- **Adopt a Developmental Approach:** Professionalism will be viewed as a continuous developmental process, with assessments designed to guide and support this development rather than merely evaluate it.

7. Professionalism Review Process

The Professionalism Review process is structured to detect and address potential issues in professionalism among students, facilitating timely and appropriate interventions. The review process involves two-tier levels; i) course/program level and ii) student professionalism committee. Periodic assessments utilizing a well-structured professionalism rubric (see appendix) and incident reports for unprofessional behaviors (see appendix) will be reviewed first by the course coordinator/program director and possibly escalated to the student professionalism committee as required (see Figure below). Professionalism will be evaluated based on the "Professionalism Performance Level G-TPCE" rubric (see appendix)

Trigger for Professionalism Review

A review is initiated under the following circumstances:

- **Low Scores on Periodic Assessment:** Scores of 3 and below in overall assessment (G-TPCE) or any professionalism domains on multiple occasions or in critical competencies.

- **Critical Incident Report:** Reports of a critical incident that may indicate a breach of professional conduct. Incident reports would be completed by faculty members following an unprofessional behavior by a student which is observed by a faculty member and/or reported by a student peer.

Professionalism Review Procedures

PreMed/PreClinical Programs:

- Course/Program Level: In premed/premedical programs, initial reports of unprofessional behavior should be managed within the course or program. This includes Minor infractions (as defined by scores of 4 on the "Professionalism Performance Level G-TPCE" rubric) handled through informal discussions and remedial guidance. For minor issues, course directors can initiate remedial actions and document these efforts. Documentation of incidents and feedback at this level should focus on guiding students toward improvement without immediate escalation.
- Professionalism Committee Level: Escalation to the committee should occur if there are repeated minor infractions indicating a pattern of unprofessional behavior and/or one incident of severe infraction Any scores of 3 or below on the "Professionalism Performance Level G-TPCE" rubric would require escalation to the professionalism committee. Escalation would involve a formal review by the Professionalism Committee.

Clinical Programs:

- Course/Program Level: In clerkships, due to the potential immediate impact on patients and the clinical learning environment, behaviors scoring 4 may be flagged for committee review by the course coordinator/program director due to the higher stakes of clinical settings. Direct observations and feedback should be more formally documented, given the potential for immediate consequences. Immediate informal feedback followed by structured remediation at the course level.
- Professionalism Committee Level: Escalation should be considered for all behaviors scoring at three or below due to the direct impact on patient safety and care. Repeated scores of 4 should also be escalated to the committee as these indicate persistent issues that have not been resolved at the course/program level. The Professionalism Committee would arrange for formal reviews and potentially more significant actions.

- Initiation of Review:
    - All reviews, whether informal or formal, are initiated by notifying the student and requiring them to engage in a structured discussion with the director of the respective course or a designated professional conduct advisor for the informal reviews or the professionalism committee for the formal reviews.
- Consultation Before Session:
    - Before the review session, the responsible director and/or committee representative would consult with the Chair of the Student Professionalism Committee and other educational leaders to understand the broader context of the student's behavior and any emerging patterns.

Decision-Making in Professionalism Trigger Review by Professionalism Committee

- Decision-Making Panel:

- All triggers will be first reviewed by the course coordinator and/or the program director. Any major incidents or unresolved minor incidents will be referred to the Student Professionalism Committee.
- The Student Professionalism Committee will consist of faculty standing members from faculty and representatives from the CMHS administration. The relevant course coordinator and/or program director would be an ad-hoc member as required. The committee may decide on no further action, a remedial program, or academic sanctions as outcomes.
- When any committee evaluates the trigger, the results for that course/clerkship will be withheld until the committee makes a decision.

- Criteria for Decision:
    - Decisions are based on a comprehensive review of the student's professionalism assessments, feedback from clinical and academic supervisors, peer evaluations, and any other relevant documentation. The committee will examine all professionalism reports available for the student, and assess professionalism according to the "Professionalism Performance Level G-TPCE" rubric".
- Outcomes of the Review:
    - No Further Action: If the panel finds no substantial issues or deems the behavior a one-off incident without serious implications.
    - Developmental Actions: For areas of concern, the student is advised to engage in specific developmental activities designed to address these issues.
    - Remedial Program: If repeated or serious breaches, the student must undertake a formal remediation program.
    - Academic Sanctions: For severe or persistent unprofessional behavior, more stringent actions, such as academic probation, failure of a component, or other penalties, may be recommended.
- Documentation and Follow-Up:
    - All discussions and decisions are meticulously documented. The student receives a copy of this documentation, which is also stored in their academic file for future reference and monitoring.
    - Regular follow-ups are scheduled to assess the student's progress with any developmental or remedial actions undertaken.

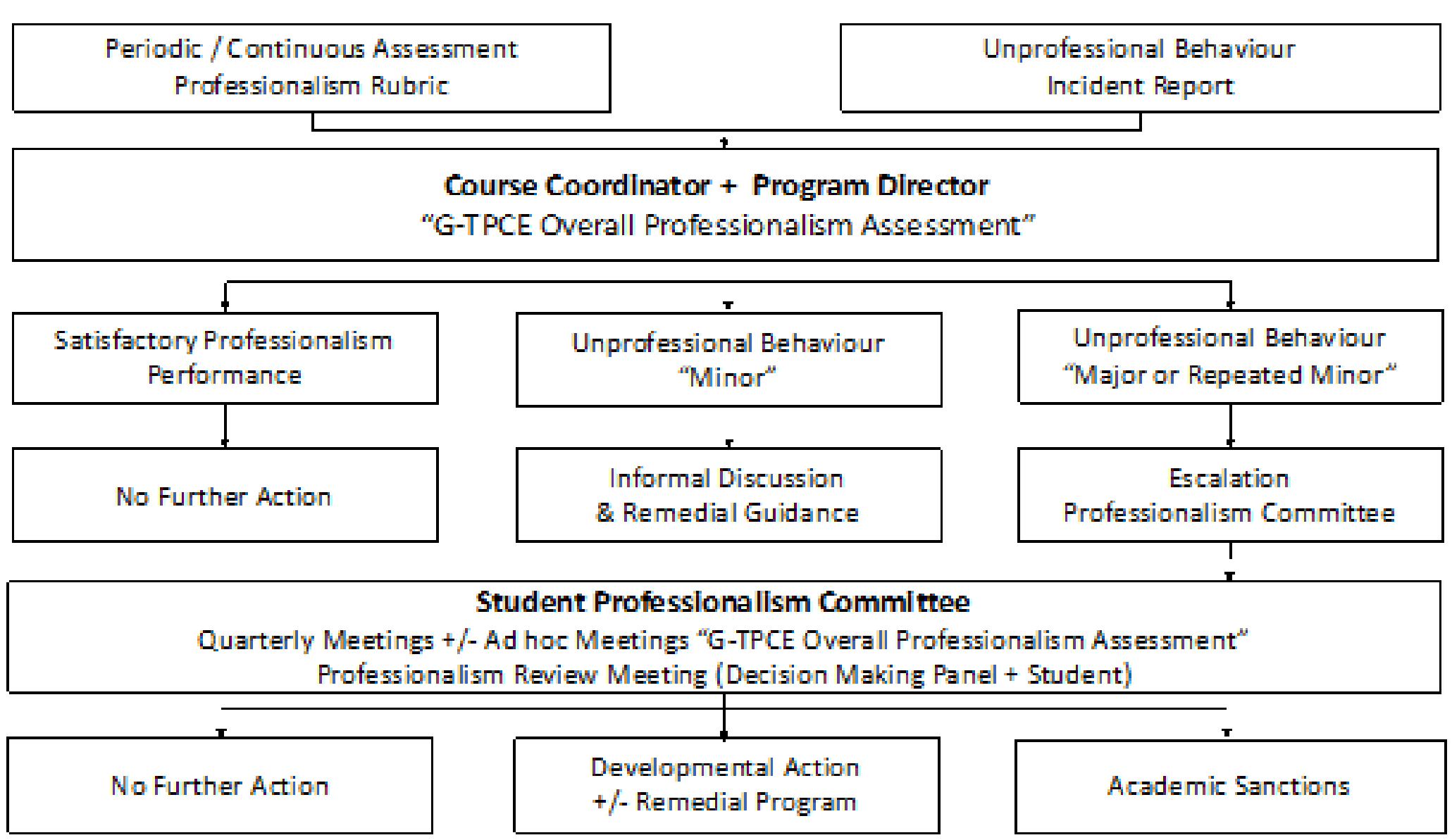

Flowchart: Unprofessional Behavior Review Process

Using the 4 I's Model to Complement Professionalism Review and Assessment

Integrating the 4 I's model into our continuous assessment and review process helps identify and address specific unprofessional behaviors in a structured manner.

Recognizing and Addressing Unprofessional Behavior Using the 4 I's Model

1. Involvement Issues (Absenteeism, Lack of Preparation, Missing Deadlines, Disengagement):
    - Action Steps:
        - For sporadic issues, initiate **informal discussions** to uncover underlying problems and offer support.
        - For persistent patterns, escalate to **formal reviews** with documentation and potential inclusion in the Professionalism Review Process.
2. Integrity Issues (Cheating, Lying, Plagiarism, Falsifying Records):
    - Action Steps:
        - At the first sign, engage in **informal inquiry** to clarify issues; educate about ethical standards.
        - For confirmed breaches, move to **formal proceedings** and potentially disciplinary actions under the Professionalism Review Process.
3. Interaction Issues (Poor Communication, Inappropriate Social Media Use, Disrespect, Harassment):
    - Action Steps:
        - Address minor issues through **informal discussions** for immediate correction.
        - Escalate severe or repeated issues to **formal reviews**, documenting and reviewing actions.
4. Introspection Issues (Lack of Self-Awareness, Poor Receptivity to Feedback):
    - Action Steps:
        - Use **reflective discussions** to improve awareness and responsiveness.
        - For ongoing concerns, consider **developmental actions** such as remediation programs or counseling.

Guiding Questions for Addressing Unprofessional Behavior

To guide the appropriate response and enhance the effectiveness of interventions, the following questions could be used when addressing unprofessional behavior:

1. What specific behavior was observed?
    - Ensure clear documentation of the behavior to understand its nature and severity.
2. When and where did the incident occur?
    - Context is crucial in influencing the appropriateness of the behavior and the response.
3. Who was involved or affected by this behavior?
    - Identify all impacted parties to address the issue comprehensively.

4. Is this the first occurrence, or is there a similar behavior pattern?
    - Determine if it's a repeated behavior requiring more significant intervention.
5. Was the behavior influenced by cultural misunderstandings or communication issues?
    - Consider if cultural factors influenced the behavior, which is particularly important in diverse environments like the UAE.
6. How did the individual react when confronted with their behavior?
    - Assess receptivity to feedback, which will guide further steps.
7. What immediate actions were taken following the observation of the behavior?
    - Note initial steps taken in response to address the behavior promptly.
8. What are this behavior's potential risks or consequences if unaddressed?
    - Evaluate the impact on the learning environment or patient care.
9. What outcome is desired or expected after addressing this behavior?
    - Define the resolution to guide actions towards a constructive outcome.
10. What support or resources are needed to address this behavior effectively?
    - Identify additional resources to address the issue, such as mediation, counseling, or training.

Conclusion

By using the 4 I's model within the broader professionalism assessment and review framework, CMHS ensures a proactive, systematic approach to managing unprofessional behavior. This approach enhances the ability to identify, address, and document issues effectively, supporting the overall development of ethical and competent medical professionals.

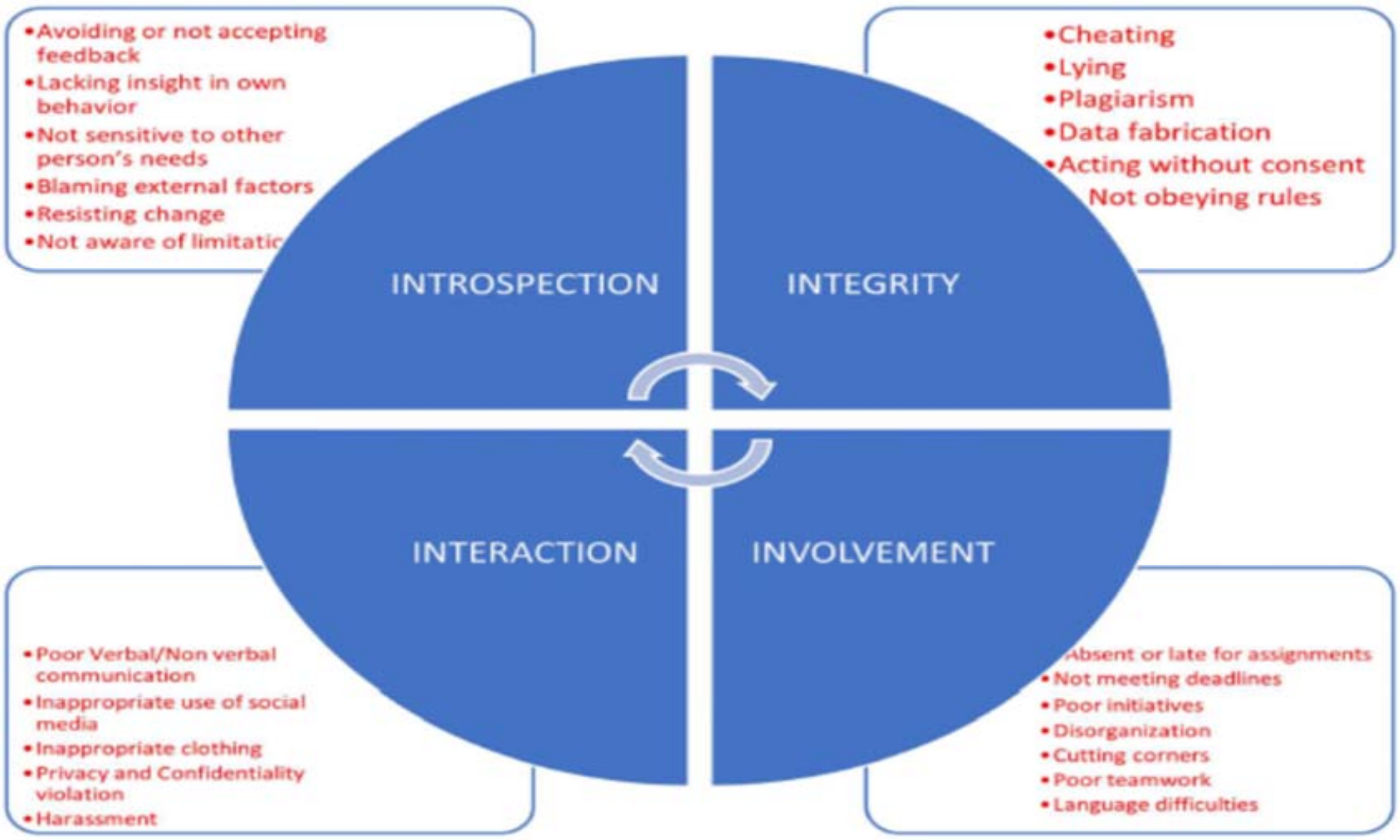

8. Processes for Addressing Unprofessional Behavior

Unprofessional behavior can undermine the educational environment and the integrity of the medical profession. To maintain high standards, the following processes are established to address such behavior effectively:

Initial Response and Reporting

- **Immediate Action**: Upon observing unprofessional behavior, the faculty or staff should initiate an informal discussion with the student to provide immediate feedback and an opportunity for self-correction.
- **Formal Reporting**: If the behavior persists or is severe, it should be formally reported to the designated authority within the college (e.g., the course director or dean's office). A detailed report should include the nature of the behavior, the context in which it occurred, and any previous attempts to address the issue (see appendix).

Formal Procedures for Managing Unprofessional Conduct

- **Investigation**: A preliminary review will determine whether further investigation is necessary once a report is filed. This may involve gathering additional information from witnesses or reviewing relevant documentation.

- **Hearing**: If a formal investigation is warranted, the student will be invited to a hearing before the Student Professionalism Committee, which will consist of standing members from faculty and representatives from the CMHS administration as well as ad-hoc members as required (e.g., relevant course coordinator and/or program director).
- **Decision**: The panel will decide the alleged unprofessional behavior based on the evidence presented. Possible outcomes include:
    - No Further Action: If the panel finds no substantial issues or deems the behavior a one-off incident without serious implications.
    - Developmental Actions: For areas of concern, the student is advised to engage in specific developmental activities designed to address these issues.
    - Remedial Program: If repeated or serious breaches, the student must undertake a formal remediation program.
    - Academic Sanctions: For severe or persistent unprofessional behavior, more stringent actions, such as academic probation, failure of a component, or other penalties, may be recommended.

Remediation Plans and Opportunities for Improvement

- **Development of Remediation Plans**: Students who demonstrate a need for improvement in professionalism will receive a tailored remediation plan. This plan will be developed by a mentor assigned by the faculty and may include specific workshops, assignments, and personal reflection activities aimed at addressing the deficiencies identified.
- **Monitoring and Assessment**: The progress of students undergoing remediation will be closely monitored through follow-up meetings and assessments to ensure they meet the objectives in their remediation plan. Based on the students' progress, adjustments to the plan may be made.
- **Completion of Remediation**: Upon successfully completing the remediation plan, the student will be reassessed to determine if the desired level of professionalism has been achieved. If so, they will resume their normal course of study; otherwise, further remedial actions may be discussed.

9. Guidelines Review and Amendments

To ensure that the Student Professionalism Guidelines remain relevant and effective, a systematic process for review and amendment is in place:

Periodic Review

- **Scheduled Reviews:** The professionalism guidelines will be reviewed every two years to ensure they remain up-to-date with the latest legal, ethical, and educational standards. The review will involve a committee of faculty, administrators, and student representatives.

- **Feedback Mechanism:** Students, faculty, and other stakeholders will be solicited for feedback during the review process. This feedback will be used to assess the guidelines' effectiveness and identify areas for improvement.

Appendix 1: Professionalism Rubric

(Adapted with Modifications from the Department of Internal Medicine's Professionalism Rubric)

Student's name:	ID:					
Professionalism Domains						
Involvement:	NA	1	2	3	4	5
Examples of Inadequate Involvement<br>- Late or absent: Unexplained absence for learning activities.<br>- Not meeting deadlines: Failure to follow the timetable and get assignments signed off.<br>- Poor initiative**:** Lack initiative / Non-assertive / Avoids patient contact / Inattentive / Non-participating / Disinterested / Lacks motivation.<br>- Attitude: Lack of commitment to teaching and learning activities and tutor meetings / Lack of engagement with clinical teams / Casual behavior.<br>- Cutting corners: Poor reliability and responsibility / Seeks minimally acceptable level of performance / Reluctance in clinical duties / Not vigilant / Avoids Work<br>- Poor teamwork: Does not function well within teams / Escaping teamwork / Disruptive with team/ Inappropriate behavior in team / Gives no feedback to others.						
Integrity:	NA	1	2	3	4	5
Examples of Inadequate Integrity<br>- Cheating-Lying: Gaining illegal access to exam materials / Influencing the teacher to get more marks / Exchanging answers during an exam / Giving false excuses when absent.<br>- Data fabrication-falsification: Fabricating the whole or part of a patient's history / Writing fake examination findings without performing it / using auto-inserted data and templates for note writing and physical status exam / Forging and recording tasks that were not performed / Writing clinical exam "normal" when did not perform / Forging signatures / Turning in work done by someone else / Allowing others to copy own work.<br>- Misrepresentation / No consent: Being introduced as "doctor" to patients / Not correcting someone who mistakes you for a physician/ No consent for clinical examination of a patient.<br>- Not obeying rules and regulations: Failing to obey rules and regulations / Failing to follow proper infection control procedures / Significant misconduct.						
Interaction:	NA	1	2	3	4	5
Examples of Inadequate Interaction<br>- Disrespectful communication by emails / writing rude or inappropriate comments / Ignoring emails or other contacts from teaching or administrative staff.<br>- Inappropriate spoken language / Using offensive language / posing provocative questions in a challenging manner/ rude or aggressive to others.<br>- Inappropriate body language with disrespect / Inappropriate behavior / Unnecessary interruptions / Dismissive or arrogant behavior<br>- Bullying / Spreading rumors / Insulting / Harassment.<br>- Lack of empathy: Insensitivity to the needs of others / Inadequate rapport with patients and families / Placing own learning above patient safety / Making a patient feel uncomfortable / Treating simulated patients as passive objects.<br>- Failure to elicit the patient's perspective: Making derogatory comments about patients / Discussing patients in public spaces or social media / Failure to respect patient confidentiality / Failure to maintain professional appearance and attire.						
Introspection:	NA	1	2	3	4	5
Examples of Inadequate Introspection<br>- Avoiding or not accepting feedback: Failure to accept responsibility for actions / Inability to incorporate feedback / Unclear expectations.<br>- Lacking insight into own behavior / Lack of self-awareness / Blaming external factors rather than skill deficiencies for bad decisions.						

- Resisting change: Diminished capacity for self-improvement / Lack of effort towards self-improvement
- Not aware of one's limitations: Acting beyond the level of competence and/or knowledge / Inappropriate advice to a patient / Giving other students inappropriate advice about clinical care / Placing own learning above patient safety.

Overall Assessment & Comments:	NA	1	2	3	4	5

Assessor Name:	Signature:	Date:

Framework for Professionalism's Overall Performance Level (G-TPCE)

Student Overall Professionalism (G-TPCE)	NA	1	2	3	4	5

5, Good: No adverse comments
4, Trace: The student had an occasional constructive or negative comment from an isolated instructor, such as "immature," but the composite evaluations and narratives were good. The occasional constructive or negative comment was mild.
3, Problem: The student had problematic comments. These comments were qualitatively serious, such as "resistant to accepting feedback," "needs continuous reminders to fulfill clerkship responsibilities," "unnecessary interruptions in class," and "inappropriate behavior in small groups both with peers and with faculty."
2, Concern: Student had problematic comments on two or more occasions at the level of “Problem”, demonstrating a longitudinal pattern of problematic professional behavior.
1, Extreme: Student has extremely problematic comments. The student received an extremely unprofessional rating based on the severity of the comment, even if only made once.

Summary Comments

Adapted with Modifications from the Department of Internal Medicine's Professionalism Rubric

Appendix 2: Illustrative Cases and Examples of unprofessional behavior

Case Study 1: Respect in Clinical Settings

Background

A medical student, Fatima, is assigned to a clinical rotation in a diverse neighborhood in Abu Dhabi. She encounters a patient, Mr. Abdullah, a recent immigrant from Egypt who speaks limited Arabic and no English. Mr. Abdullah appears anxious and uncomfortable, making it difficult to conduct a routine examination.

Observation

Fatima notices that Mr. Abdullah hesitates when she attempts to communicate directly with him. She recalls her training on cultural competence, particularly the importance of respect and understanding in patient interactions within the UAE's multicultural setting.

Action Taken

- **Cultural Sensitivity Displayed:** Fatima gently asks Mr. Abdullah if he would be more comfortable communicating in his native dialect or if he would prefer to have a family member present to help translate.
- **Involvement of a Cultural Mediator:** Upon understanding that Mr. Abdullah prefers speaking in his native dialect, Fatima arranges for a colleague who speaks Egyptian Arabic fluently to assist in the consultation.
- **Patient-Centered Approach:** She ensures that Mr. Abdullah's cultural and personal preferences are respected during the examination, using respectful body language and allowing extra time for him to express his concerns.

Outcome

Mr. Abdullah visibly relaxes and cooperates fully with the examination. He expresses gratitude for Fatima's consideration and respect for his cultural background. Fatima's supervisor praises her for her exemplary sensitivity and patient-centered approach.

Lessons Learned

This case illustrates the importance of cultural sensitivity and respect in clinical settings, especially in the UAE's diverse society. By recognizing and acting on the patient's discomfort, Fatima demonstrated professionalism and enhanced the quality of care provided.

Case Study 2: Integrity in Academics

Background

During a major examination, Amna, a third-year medical student, notices two of her peers whispering and exchanging notes — clear violations of the exam rules. She is torn between reporting the incident and staying silent, fearing peer backlash.

Observation

Amna observes the cheating continue for several minutes. She recalls the emphasis on integrity and honesty in her professional development classes and recognizes that not reporting could undermine the fairness of the examination process.

Action Taken

- **Seeking Guidance:** Initially unsure, Amna decides to seek advice without revealing the students' identities. After the exam, she speaks to her course director to discuss her observations and her dilemma.
- **Anonymous Reporting:** Following the discussion, Amna chooses to report the incident anonymously, providing enough detail to allow for a proper investigation, aligning with the college's policies on academic integrity.
- **Supportive Environment:** The faculty ensures confidentiality and supports Amna, emphasizing the importance of her integrity without putting her in direct conflict with her peers.

Outcome

The investigation confirms the cheating, and the students are subjected to appropriate remedial actions. Amna receives support from her faculty, reinforcing that her decision to uphold integrity was correct.

Lessons Learned

This case underscores the critical role of integrity in academics and the importance of individual action in maintaining ethical standards. It also highlights the support systems for students who report unprofessional behavior, ensuring they can act without fear.

Case Study 3: Digital Professionalism

Background

Khalid, a fourth-year medical student, shares a humorous but inappropriate meme about patient interactions with classmates in a private social media group. The meme is accidentally leaked outside the group and quickly spreads, causing distress among patients and faculty who see it.

Observation

The shared meme reflects poorly on the students' professionalism and potentially damages the trust between patients and the medical community.

Action Taken

- **Immediate Acknowledgment:** Once aware of the situation, Khalid immediately recognizes his mistake and the potential harm it could cause.
- **Apology and Reflection:** He sincerely apologizes to the group and seeks advice from his mentor on how to handle the situation publicly.
- **Corrective Actions:** Khalid works with the college's digital professionalism committee to craft a public apology and participates in a digital professionalism and ethics workshop.
- **Proactive Learning:** He volunteers to lead a student seminar on the risks and responsibilities of social media use as a healthcare professional.

Outcome

Khalid's prompt acknowledgment and willingness to learn from his mistake mitigates the negative impact. His actions turn a potential disciplinary issue into a learning opportunity for the entire student body.

Lessons Learned

This case illustrates the importance of digital professionalism and the impact of social media on personal and professional reputation. It demonstrates the need for careful consideration before sharing content and the positive outcomes that can arise from taking responsibility for one's actions in the digital sphere.

Examples of unprofessional behavior

Answering Phone Inappropriately

Scenario: During a clinical rotation, a medical student repeatedly takes personal phone calls in the presence of patients, ignoring the clinical duties and the privacy and comfort of the patient.

Disrespect

Scenario: A student speaks over peers and instructors during discussions, dismissively arguing points without listening to others' views. This lack of respect for the contributions of colleagues and educators undermines the collaborative learning environment.

Lack of Altruism

Scenario: A student refuses to help peers with shared responsibilities or learning opportunities, prioritizing personal interests over the common good and the welfare of others, including patients.

Cheating

Scenario: During a critical exam, a medical student, concealed by the privacy of a cubicle, uses a smartwatch to access test answers. This behavior not only violates the academic integrity of the institution but also compromises the student's future ability to perform independently in high-stakes environments, such as in patient care settings.

Plagiarism

Scenario: A medical student submits a research paper that extensively copies work from published sources without proper citation. The paper is meant to demonstrate the student's own research capabilities and understanding of the subject matter.

Falsification

Scenario: In a clinical setting, a student records blood pressure measurements that were never taken to complete a patient chart quickly. This action is driven by pressure to manage time efficiently, but it fabricates crucial patient data.

Inappropriate Use of Social Media

Scenario: A student posts confidential information about patients or disrespectful comments about classmates or faculty on social media platforms.

Misuse of Email or Messaging Platforms

Scenario: A student sends unprofessional or offensive emails to faculty or uses messaging platforms to harass peers.

Unauthorized Access or Sharing of Sensitive Information

Scenario: A student accesses or shares restricted academic content, such as exam materials, or patient information without authorization.

Punctuality

Scenario: A student consistently arrives late to classes, clinical rotations, or meetings with faculty members.

Attendance

Scenario: A student frequently misses classes, clinical rotations, or mandatory events without valid reasons or proper notifications.

Presence

Scenario: During important educational or clinical activities, a student is physically present but regularly disengaged, often distracted by non-related tasks such as browsing the internet on their phone.

Dress Code Non-compliance

Scenario: A student repeatedly ignores the dress code for clinical rotations, wearing inappropriate or unclean attire.

Unpreparedness

Scenario: A student consistently fails to prepare for classes or clinical rotations, unable to contribute or perform required tasks.

Appendix 3: Unprofessional Behaviour Report Form

This form is intended to address situations where a student has put a patient or someone else at significant risk because of their behavior or situations in which a student has engaged in conduct unbecoming of a physician in training.

A: Student Identification:

Student name	
University ID	
Date of incident	
Course/Clerkship/Component/Other at the time of the incident	

B: Area (s) of concern (Check all that apply; See the professionalism rubric for details):

Integrity

Interaction

Introspection

Involvement

C: Provide details of the incident. (Please provide as much detail as possible and attach any documentary evidence, if applicable.)

Observation (Describe as objectively as possible, with exact details of words or actions).

Thoughts: What did the faculty or observer think about the behavior?

Feelings: What feelings were caused by the behavior?

Recommendations: What needs to be changed or done in the future?

D: Submitted by:

Name	
Role	
University ID, if applicable	
Date Submitted:	

Appendix 4: Cultural Sensitivity

Hierarchy and Respect

Respect for Authority: In UAE culture, there is a strong emphasis on respecting authority and hierarchy. The guidelines should reflect this respect, emphasizing the importance of students properly respecting faculty, staff, and senior medical professionals.

Faculty-Student Dynamics: Encourage faculty to be approachable while maintaining the cultural norm of respect for their position. This can help create an environment where students feel comfortable seeking advice or help.

Communication Styles

Indirect Communication: In many Middle Eastern cultures, indirect communication is common as it helps maintain harmony and face-saving. Encourage faculty and students to be clear yet respectful, avoiding overly direct or blunt feedback that might be perceived as disrespectful.

Nonverbal Communication: Nonverbal cues are highly significant. Encouraging awareness of body language, personal space, and other nonverbal signals in professional interactions can help avoid misunderstandings.

Conservatism and Modesty

Dress Code: Professional attire should be clearly defined, respecting Islamic principles of modesty. This is particularly important in clinical settings where frequent interactions with patients from diverse backgrounds occur.

Gender Interactions: Guidelines should address gender interactions sensitively, respecting cultural norms regarding physical contact and communication between opposite genders.

Religious Observances

Prayer Times: Acknowledge and accommodate the practice of praying five times a day, which might require scheduling flexibility for Muslim students.

Ramadan and Fasting: During Ramadan, consider the impact of fasting on students' energy levels and concentration. Adjusting academic and clinical schedules to accommodate shorter working hours this month can be considered.

Family Involvement

Family Engagement: In the UAE, family plays a central role in decision-making. Professionalism guidelines might consider how students' families are involved in their educational journey, particularly in scenarios requiring disciplinary actions or support services.

Local Legal and Ethical Standards

UAE Laws and Ethics in Medicine: Professionalism guidelines must align with UAE laws and ethical standards in healthcare, which might differ from Western standards. This includes laws on patient privacy, medical reporting, and treatments allowed under local regulations.

Cultural Sensitivity Training

Incorporate Training Modules: Develop modules specifically aimed at understanding and navigating the multicultural environment of the UAE. This training can help students and faculty from diverse backgrounds interact more effectively and respectfully with each other and patients.

Appendix 5: Bibliography

- Mak-van der Vossen M, Teherani A, van Mook W, Croiset G, Kusurkar RA. How to identify, address, and report students' unprofessional behavior in medical school. *Med Teach*. 2020;42(4):372-379. doi:10.1080/0142159X.2019.1692130
- Hodges BD, Ginsburg S, Cruess R, et al. Assessment of professionalism: recommendations from the Ottawa 2010 Conference. *Med Teach*. 2011;33(5):354-363. doi:10.3109/0142159X.2011.577300
- Cruess SR, Cruess RL, Steinert Y. Supporting the development of a professional identity: General principles. *Med Teach*. 2019;41(6):641-649. doi:10.1080/0142159X.2018.1536260
- Abdel-Razig S, Ibrahim H, Alameri H, et al. Creating a Framework for Medical Professionalism: An Initial Consensus Statement From an Arab Nation. *J Grad Med Educ*. 2016;8(2):165-172. doi:10.4300/JGME-D-15-00310.1
